# Phase II multicentre study of docetaxel plus cisplatin in patients with advanced urothelial cancer

**DOI:** 10.1038/sj.bjc.6600121

**Published:** 2002-02-01

**Authors:** X García del Muro, E Marcuello, J Gumá, L Paz-Ares, M A Climent, J Carles, M Sánchez Parra, J L Tisaire, P Maroto, J R Germá

**Affiliations:** Institut Català d'Oncologia, Department of Medical Oncology, Avda Gran Vía km 2.7, 08907 L'Hospitalet, Barcelona, Spain; Hospital de Sant Pau, Antonio M Claret 167, 08025, Barcelona, Spain; Hospital de Sant Joan, President Companys s/n, 43201. Reus, Tarragona, Spain; Hospital Doce de Octubre, Av Córdoba Km 5.4 28041, Madrid, Spain; Instituto Valenciano de Oncología, Profesor B Báguena 19, 46009, Valencia, Spain; Hospital del Mar, P Marítimo 25, 08003, Barcelona, Spain; Hospital de Aránzazu, Dr Beguiristain s/n. 20014, San Sebastián. Guipuzcoa, Spain; Medical Department, Aventis Pharma SA, 28027, Madrid, Spain

**Keywords:** urothelial cancer, docetaxel, cisplatin, bladder carcinoma

## Abstract

A multicentre phase II trial was undertaken to evaluate the activity and toxicity of docetaxel plus cisplatin as first-line chemotherapy in patients with urothelial cancer. Thirty-eight patients with locally advanced or metastatic transitional-cell carcinoma of the bladder, renal pelvis or ureter received the combination of docetaxel 75 mg m^−2^ and cisplatin 75 mg m^−2^ on day 1 and repeated every 21 days, to a maximum of six cycles. The median delivered dose-intensity was 98% (range 79–102%) of the planned dose for both drugs. There were seven complete responses and 15 partial responses, for and overall response rate of 58% (95% CI, 41–74%). Responses were even seen in three patients with hepatic metastases. The median time to progression was 6.9 months, and the median overall survival was 10.4 months. Two patients who achieved CR status remain free of disease at 4 and 3 years respectively. Grade 3–4 granulocytopenia occurred in 27 patients, resulting in five episodes of febrile neutropenia. There was one toxic death in a patient with grade 4 granulocytopenia who developed acute abdomen. Grade 3–4 thrombocytopenia was rare (one patient). Other grade 3–4 toxicities observed were anaemia (three patients), vomiting (five patients), diarrhoea (four patients), peripheral neuropathy (two patients) and non-neutropenic infections (seven patients). Docetaxel plus cisplatin is an effective and well-tolerated regimen for the treatment of advanced urothelial cancer, and warrants further investigation.

*British Journal of Cancer* (2002) **86**, 326–330. DOI: 10.1038/sj/bjc/6600121 www.bjcancer.com

© 2002 The Cancer Research Campaign

## 

Chemotherapy is the treatment of choice for patients with locally advanced and metastatic urothelial cancer. The combination of methotrexate, vinblastine, doxorubicin and cisplatin (M-VAC) has been the most widely used regimen, with reported response rates of 36 and 78% (Sternberg *et al*, 1989; Saxman *et al*, 1997). Long-term results of the Phase III Intergroup Study showed that the treatment with M-VAC provided a significant survival advantage over cisplatin alone (Saxman *et al*, 1997). Moreover, after a major response to chemotherapy, a small number of patients (4.3%) remained free of disease after long-term follow-up. This percentage of long-term survivors may be increased when postchemotherapy surgery or radiotherapy is performed in selected responding patients (Fossa *et al*, 1996; Dodd *et al*, 1999). The data showed that urothelial cancer is a disease sensitive to chemotherapy. However, its long-term results are still poor and its toxicity is substantial. Therefore, in recent years the necessity arose to identify new drugs and schedules that were more active and tolerable than the ones that were currently being used.

Docetaxel is a wide spectrum chemotherapeutic agent that acts by promoting and stabilizing the assembly of microtubules, resulting in the inhibition of cellular division. In phase II trials, it has shown activity against advanced bladder carcinoma (McCaffrey *et al*, 1997; de Wit *et al*, 1998). Cisplatin has been considered the principal agent in the treatment of urothelial cancer. The objective of this study is to evaluate the activity and toxicity of the combination of docetaxel and cisplatin in first-line treatment of advanced urothelial cancer.

## MATERIALS AND METHODS

### Patients

Patients with histological confirmation of metastatic or locally advanced (T4b, N2-3) transitional-cell carcinoma of the bladder, renal pelvis or ureter, not curable with surgery, were eligible. Patients with mixed tumours including transitional-cell carcinoma were considered eligible, whereas those with pure squamous, adenocarcinoma, or small-cell carcinoma were not. Patients must not have received prior chemotherapy for advanced disease, although prior adjuvant or neoadjuvant chemotherapy was allowed if this was completed more than 6 months before study entry. Patients were required to have bidimensional measurable disease and no previous radiotherapy of the indicator lesion. Patients were also required to be 18 years or older, with a Karnofsky performance status of 60 to 100. Other inclusion criteria were as follows: normal baseline haematologic parameters, creatinine clearance of 60 ml min^−1^ or more, a normal bilirubin level, a alkaline phosphatase level of less than six times the upper normal limit, and transaminase levels of less than 3.5 times the upper normal limit or less than 1.5 times in case of association with alkaline phosphatase greater than 2.5 times the norm. Patients with known CNS metastases, pre-existing grade 1 peripheral neuropathy, history of prior malignancy, or significant cardiac disease were not eligible for this study. Written informed consent was obtained from all patients before study entry. The study was carried out with ethical committee approval at each participating hospital.

### Treatment schedule

Docetaxel was administered at a dose of 75 mg m^−2^, diluted in 250 ml of 5% glucose, as a 1 h infusion. Cisplatin 75 mg m^−2^ was infused in 500 ml of normal saline over 30–60 min, with adequate pre- and post-hydration and mannitol. Both drugs were given on day 1 and repeated every 3 weeks. Premedication included dexamethasone, 8 mg orally b.i.d., the day before and four consecutive days following chemotherapy. Antiemetic treatment consisted of intravenous ondansetron or granisetron in combination with dexamethasone 20 mg on day 1. Cycles were not started unless the granulocyte count was >1500 mm^−3^ and platelets were >100 000 μl^−1^. Prophylactic use of growth factors (G-CSF) was not routinely recommended. However, if grade 4 granulocytopenia or febrile neutropenia was present, prophylactic Lenograstim, 263 μg day^−1^ over 10 days, was administered in subsequent cycles. The docetaxel dose was reduced to 55 mg m^−2^ if patients experienced grade 4 thrombocytopenia, febrile neutropenia despite prophylactic administration of G-CSF, or grade 2 hepatotoxicity. Doses of both agents were reduced by 25% if patients experienced grade 2 peripheral neuropathy. Patients were taken off the study if creatinine clearance decreased to less than 50 ml min^−1^. Patients received treatment for a maximum of six cycles unless they developed progressive disease or experienced excessive toxicity as judged by the investigator or the patient.

### Outcome evaluation

National Cancer Institute common toxicity criteria were used to analyze toxicity. Response evaluation was performed every three cycles using standard WHO criteria. Even patients receiving just one course were considered evaluable for response and toxicity assessment. Patients were followed for survival and disease progression every 3 months until death or loss to follow-up. The method of Kaplan–Meier was used to estimate duration of response, time to progressive disease and overall survival. The planned sample size was 35 patients, using a two-stage sequential design, with the assumption that the regimen would not be of interest if it had a response rate of less than 30% but would be of considerable interest if it had a response proportion of 50% or more (Fleming, 1982).

## RESULTS

From February 1997 to August 1999, 38 patients were entered into the study at seven centres. All patients were eligible for the study, and all assessable for toxicity and response. Pre-treatment characteristics of the patients are summarized in Table 1

**Table 1:**
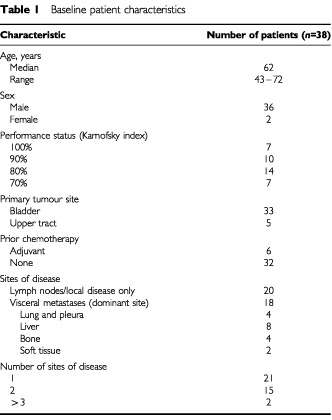
Baseline patient characteristics

. Twenty (53%) patients had locally advanced or metastatic lymph node disease, and the remaining 18 (47%) had metastatic visceral or soft tissue disease. Six patients had received prior systemic adjuvant chemotherapy with cisplatin or carboplatin-containing regimens.

A total of 166 cycles of chemotherapy were administered. The median number of cycles received per patient was six (range, 1–6 completed cycles). Of the 38 patients, 35 (92%) received at least two cycles of chemotherapy. The median delivered dose-intensity was 98% (range, 79–102%) of the planned dose for both drugs. Fifteen patients received G-CSF in a total of 48 cycles of chemotherapy. G-CSF was administered prophylactically in 42 cycles, and as a treatment of febrile neutropenia in six cycles. Overall, patients were ultimately withdrawn from therapy due to progressive disease (nine patients), adverse events (six patients), stable disease (three patients), or request (one patient). Twenty-two of the 38 assessable patients obtained an objective response, for an overall response rate of 58% (95% confidence interval (CI), 41–74%), with seven patients obtaining a radiological complete response (CR, 18%) and 15 a partial response (PR, 39.5%). An additional four patients (10.5%) had stable disease, and 11 patients had progressive disease. Responses were seen in three out of the six patients who had received prior adjuvant chemotherapy. Fifteen of the 22 patients who achieved a response had locally advanced or metastatic lymph node disease exclusively. However, five patients had visceral metastases, including three with liver and two with lung disease, and two additional patients had soft tissue metastases. The median duration of response was 10.5 months (95% CI, 7.6 to 16.8). The median time to progressive disease was 6.9 months (95% CI, 6.2 to 10.5), and the median overall survival time for all patients was 10.4 months (95% CI, 6.3 to 17.4 months). At the time of the report, two patients who had achieved CR status remain alive and free of recurrence at 48 and 36 months respectively. Both had exclusively locally advanced and lymph node disease before the chemotherapy. One of them underwent salvage cystectomy after chemotherapy, and no viable tumour was found at pathological study.

Table 2

**Table 2:**
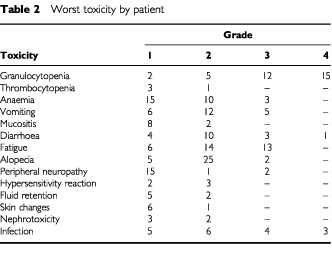
Worst toxicity by patient

lists the maximum grade of common toxicities observed in each patient. Grade 3 and 4 toxicities were primarily haematological. Twenty-seven (71%) patients experienced grade 3 or 4 granulocytopenia; however, only five (13%) patients in five of the total cycles experienced febrile neutropenia. Three patients developed grade 3 anaemia. Clinically significant thrombocytopenia was observed in one patient. There was one toxic death in a patient with grade 4 granulocytopenia who developed acute abdomen. Grade 3 and 4 nonhaematologic toxicity was experienced by five (13%) patients with nausea and vomiting, four patients (10%) with diarrhoea, two patients (5%) with peripheral neuropathy, and one patient with renal toxicity. A total of seven patients had severe non-neutropenic infections, that consisted of four urinary tract infections, two pneumonia, and one candida lung abscess in a patient with concurrent corticoesteroid treatment. One patient died on study due to gastrointestinal haemorrhage without thrombocytopenia. Two patients experienced episodes of cardiac arrhythmias, and one patient had a myocardial infarction. Most patients had some degree of fatigue with this therapy that did not lead to dose modification. Hair loss was common and 27 (71%) patients had total alopecia. Infusion-related hypersensitivity reactions were seen in five patients and all were mild (grade 1 and 2). Severe bronchospasm was not observed. Grade 1 and 2 fluid retention occurred in five and two patients respectively, and it was generally noted after four or five cycles of treatment.

## DISCUSSION

The initial experience with docetaxel in the treatment of urothelial cancer demonstrated single-agent activity in a series of patients previously treated with chemotherapy (McCaffrey *et al*, 1997). One trial performed on 30 chemotherapy-naive patients showed a response rate of 31% (de Wit *et al*, 1998), suggesting that docetaxel could be among the drugs with high activity against urothelial cancer. In addition, docetaxel can be administered safely to patients with impaired renal function (Dimopoulos *et al*, 1998), a condition frequently associated with bladder carcinoma.

The present study evaluated docetaxel in combination with cisplatin which is generally considered to be the most active agent against urothelial cancer. Prior phase I studies showed the feasibility of this combination and its activity on different tumours (Pronk *et al*, 1997). The response rate observed for urothelial cancer in our study is within the range of the responses seen with other conventional (Sternberg *et al*, 1989) and newer schedules (Von der Maase *et al*, 1999; Dreicer *et al*, 2000) which are considered highly active. Responses were even seen in patients with visceral metastases, traditionally considered resistant to M-VAC chemotherapy, and also in patients previously treated with adjuvant chemotherapy, where the proportion of responses was similar to the whole group. It should be noted that two patients in our series have achieved long-term survival. At the time of the report, they remained alive and free of disease, at 4 and 3 years from the treatment.

The toxicity of this regimen was generally acceptable and manageable. The most common toxicity was haematological, mainly granulocytopenia. However, most episodes of granulocytopenia were brief and did not cause clinical repercussion, since only 13% of the patients experienced febrile neutropenia. Nevertheless, there was a toxic death associated with acute abdomen and severe granulocytopenia. This rare and serious complication has been reportedly associated with different chemotherapy schedules, including docetaxel (Cardenal *et al*, 1996). Also, several episodes of serious non-neutropenic infections were observed. They were probably linked to comorbidities and complications due to the neoplasm rather than to the treatment itself. In spite of the potential neurotoxicity of cisplatin and docetaxel, there was not a significantly high incidence of severe peripheral neuropathy. The incidence of thrombocytopenia and mucositis, two complications that may cause important morbidity and are frequently seen with other common regimens (Von der Maase *et al*, 2000), was very low.

Recently, two studies that used the same regimen in bladder carcinoma have been reported (Sengelov *et al*, 1998; Dimopoulos *et al*, 1999) (Table 3

**Table 3:**
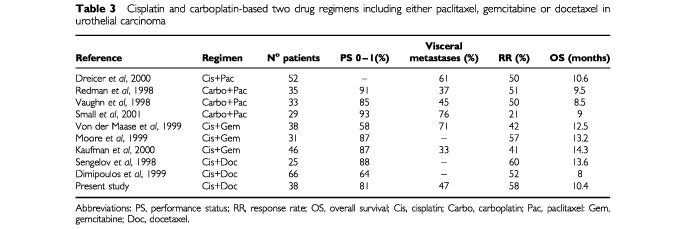
Cisplatin and carboplatin-based two drug regimens including either paclitaxel, gemcitabine or docetaxel in urothelial carcinoma

). The results of these three studies confirm the efficacy of this regimen. Variations in the distribution of pre-treatment prognostic features and the multicentre nature of two studies could justify the differences in the results between them. The toxicity profile observed was similar in the three studies, the side effects being generally of mild to moderate intensity.

Two additional studies have evaluated docetaxel in other combinations in the treatment of urothelial cancer. One of them evaluated the three-drug combination with cisplatin and epirubicin (Pectasides *et al*, 2000). The response rate was 66.7% and the median overall survival was 14.5 months. Nevertheless, more than half of the patients required dose reductions due to haematological toxicity, despite the frequent use of G-CSF. The other study assessed the association of docetaxel and ifosfamide after failure of cisplatin-based chemotherapy (Krege *et al*, 2001). This combination showed activity with acceptable tolerability.

In the last few years, several clinical trials aimed at identifying polychemotherapy regimens with new drugs active against urothelial cancer have been initiated. The objective has been to find combinations that demonstrate improved efficacy and a better toxicity profile compared with that of M-VAC. The most extensively studied drugs have been gemcitabine (Moore *et al*, 1999; Von der Maase *et al*, 1999; Kaufman *et al*, 2000) and paclitaxel (Redman *et al*, 1998; Vaughn *et al*, 1998; Zielinski *et al*, 1998; Dreicer *et al*, 2000; Small *et al*, 2000), each in combination with cisplatin or carboplatin. These studies have usually shown an elevated activity with a favourable toxicity profile (Table 3). However, comparing results of different phase II trials is unreliable, because they are very dependent on the prognostic features of the patients included in the trials (Bajorin *et al*, 1999). At the moment, there is only one phase III trial available comparing gemcitabine plus cisplatin to standard M-VAC (Von der Maase *et al*, 2000). Four hundred and five patients were included, and no differences in activity in both regimens were found. However, the tolerance profiles were different, showing a significantly lower incidence of serious granulocytopenia and mucositis in the patients treated with gemcitabine plus cisplatin.

A few trials with three-drug regimens containing two new drugs have also been performed in recent years. The combination of paclitaxel, gemcitabine and cisplatin (Bellmunt *et al*, 2000); the one with ifosfamide, paclitaxel and cisplatin (Bajorin *et al*, 1998); and one with paclitaxel, gemcitabine and carboplatin (Hussain *et al*, 2001) showed promising activity with predominantly haematological toxicity. The last study was comprised of patients with normal and poor renal function (Hussain *et al*, 2001). The association of paclitaxel, vinblastine and cisplatin, however, resulted in a poor efficacy (Mulatero *et al*, 2000). Another new approach studied, with preliminary encouraging results, consisted of a sequential schedule with doxorubicin and gemcitabine followed by ifosfamide, paclitaxel and cisplatin (Dodd *et al*, 2000).

In summary, the present study shows that the association of docetaxel and cisplatin is an effective regimen in the treatment of advanced urothelial cancer. The results also show that a small number of patients, who would otherwise succumb to disease, can achieve long-term disease-free survival after this chemotherapy regimen. Although the toxicity of this regimen was not insignificant, it was tolerable considering that the severe toxicity observed was in general reversible and manageable. These results, added to the other recent studies that included docetaxel in their treatment regimens, indicate the interest in this drug in the treatment of urothelial cancer as an alternative to conventional therapeutic regimens. Further studies aimed at determining more accurately the potential of docetaxel are warranted together with investigation into new strategies that will introduce it into three-drug regimens or in combinations without cisplatin.
